# Genetic architecture and genomic prediction of vase life in carnation

**DOI:** 10.3389/fpls.2025.1673111

**Published:** 2025-12-05

**Authors:** Hugo H. Tavera, Dominik Losert, Maike Boxriker, Robert Boehm, Carola Zenke-Philippi

**Affiliations:** 1 Institute of Agronomy and Plant Breeding II, Justus Liebig University, Giessen, Germany; 2 Klemm + Sohn GmbH & Co KG, Stuttgart, Germany

**Keywords:** carnation, vase life, genetic architecture, QTL analysis, GWAS, genomic selection

## Abstract

Vase life is a key trait for carnation cut flowers. A better understanding of the genetic basis of vase life is needed to implement selection methods like marker-assisted selection or genomic selection. Our objective was to investigate the genetic architecture of vase life and evaluate the potential of including known QTL in genomic prediction models to improve prediction accuracy. We constructed a linkage map from two segregating F1 carnation populations based on 5,412 SNP markers. Quantitative trait loci analysis detected one QTL for each of the two populations. The QTL were located on chromosomes 10 for population 1 and chromosome 11 for population 2 and accounted for 2.84 and 5.09% phenotypic variation, respectively. A genome-wide association study revealed potential genomic regions of interest for vase life. All detected markers accounted individually for less than 6% of phenotypic variation and were spread across ten chromosomes. This suggests that vase life is a polygenic trait. We conducted a cross-validation study in which 1 through 120 top scoring SNP markers were fitted as fixed effects to the baseline rigde-regression best unbiased linear prediction model. Fitting 1 to 50 top scoring SNP markers improved prediction accuracy compared to the baseline model by 0.016 on average. Fitting the 25 top scoring SNP markers resulted in the greatest prediction accuracy improvement, from 0.75 to 0.78. These findings extend the knowledge on the genetic basis of vase life and attest to the potential of genomic selection for breeding cut carnation with longer vase life.

## 1 Introduction

Carnation (*Dianthus carophyllus* L.) is among the economically most important ornamental species worldwide (Onozaki and Yagi, 2020). A large portion of cut carnation production takes place in the Southern hemisphere, mainly South and Central America as well as East Africa, while the biggest markets for it are in North America, Europe and Asia. This means that cut flower longevity, which is determined by both transportability and vase life, is critically important due to the increasing reliance on longer commercialization routes as well as the influence of vase life in customer satisfaction (Onozaki et al., 2001). Vase life is defined as the time span between placing a cut flower in water until the loss of its ornamental value. This time span encompasses many physiological aspects of the plant, like opening of the flowers, loss of water, senescence and abscission of plant organs, changes in color of petals and leaves and bending of the stem, among others (Mibus, 2018). These different aspects contribute to the complexity of vase life as a trait.

Molecular markers have been developed for the selection of carnation varieties with longer vase life (Boxriker et al., 2017a). Nevertheless, as is the case for most ornamental species, breeding programs for the improvement of vase life in carnation still rely mainly on morphological markers and conventional breeding methods (Onozaki, 2018; Tanase, 2020a). A better understanding of the genetic basis of vase life could facilitate the implementation of other types of selection strategies for this trait, like marker-assisted selection or genomic selection. Quantitative trait loci (QTL) analyses and genome-wide association studies (GWAS) are key tools to study the genetic architecture of complex traits (Holland, 2007). An essential part of QTL analyses is the availability of a genetic linkage map to precisely locate QTL on the target genome. Genetic linkage maps have been constructed for carnation to study economically relevant traits through QTL analysis (Yagi et al., 2012, 2017, 2020). With the advent of next-generation sequencing, availability of genetic markers has significantly increased, even for non-major crops. This, together with the development of bioinformatic tools for genetic map construction and QTL analysis for outcrossing populations, has facilitated the study of complex traits in perennial species, like carnation (Margarido et al., 2007; Gazaffi et al., 2014; Taniguti et al., 2023). QTL analysis has been sucessfully implemented in carnation to identify DNA markers linkend to bacterial wilt resistance and flowering time (Yagi et al., 2020). Despite the relevance of vase life for transportabilty and product quality of cut carnation, no QTL analysis studies have focused on this trait to date (Katoch et al., 2024).

Genomic selection has been shown to increase genetic gain in both animal and plant breeding (Bernardo and Yu, 2007; Meuwissen et al., 2016; Crossa et al., 2017). It is particularly effective for traits with a polygenic genetic architecture, where variation is controlled by multiple loci with small effects spread throughout the genome (Bernardo, 2008; Heffner et al., 2010). In chrysanthemum, another economically important ornamental species, genomic prediction has been implemented for polygenic traits like plant height and flowering time (Zhang et al., 2024; Su et al., 2024). For these traits, the reported prediction accuracy, measured as the correlation between predicted and observed values, was 0.5 and 0.53, respectively. Additionally, these authors performed GWAS-assisted genomic prediction by including top associated SNP markers into their genomic prediction model, with the added SNP markers explaining between 0.5-8.01% of the phenotypic variation. This approach increased prediction accuracy for both traits by an average of 0.38. Improvement in prediction accuracy by including known QTL as fixed effects has been reported in other experimental as well as simulation studies (Bernardo, 2014; Spindel et al., 2016; Rice and Lipka, 2019; Sehgal et al., 2020). In perennials, which include many commercially important ornamental species, genomic selection can be especially beneficial, due to its potential to shorten generation intervals (Voss-Fels et al., 2019; Seyum et al., 2022). Efforts to generate cut carnation varieties with longer vase life have been carried out (Onozaki et al., 2001, 2011). Cross-breeding and selection of lines with long vase life were conducted over seven generations, from 1992 to 2008, where lines with long vase life were selected each generation and used as parents for the next generation (Onozaki, 2018). Through these efforts, the mean vase life had an net increase of 8.5 days, from 7.4 in the first generation to 15.9 days in the last generation. Although effective, conventional cross-breeding techniques can take decades to produce new long vase life varieties (Onozaki and Yagi, 2020). Genomic selection has the potential to reduce time and costs for breeding programs focused on prolonging vase life in cut carnation.

The recent publication of the first chromosome-scale genome assembly for cultivated carnation helps integrate genomic tools for comprehensive analysis of the genetic basis of vase life in carnation (Zhang et al., 2022). For this study, two experimental F1 crosses segregating by vase life were used to investigate vase life in cut carnation (Boxriker et al., 2017a, b). The objectives of this study were: (1) to build a genetic linkage map for carnation, (2) investigate the genetic architecture of the trait vase life by conducting QTL analysis and GWAS, and (3) to evaluate the potential of genomic prediction for vase life in carnation and the integration of SNP markers detected by QTL analyis or GWAS in genomic prediction models.

## 2 Materials and methods

### 2.1 Phenotypic data

Vase life measured in days from a two-phase one-year trial was obtained from Boxriker et al. (2018). The entirety of the trial is described in detail in Boxriker et al. (2017b). Briefly, the trial’s first phase took place in the greenhouse, where 556 carnation genotypes were cultivated in a resolvable row-column design. Surface boxes (Beekenkamp, Netherlands) were defined as rows and the nine positions within the boxes were defined as columns. Each genotype had four complete replications. After reaching the flower development stage, before blooming, two stems per replicate of each genotype were harvested. For the second phase, lower leaves were removed, the stems were trimmed to 50 cm and put in fresh tap water in order to assess vase life. The level of water was maintained constant by refilling it periodically. Ambient conditions were mantained constant: 12 hour photoperiod of 800 lx provided by LED lamps and an average daily temperature of 20.1 ± 0.5°C. An air exchange rate of 1.5 air changes per hour was maintained by the air conditioning system. Once in the second phase, each flower was checked daily for symptoms of senescence like rolling-in, fading or browning of the petal edges, as well as wilting and bent necks of the stems. The vase life of each flower was recorded as the number of days before the manifestation of the mentioned symptoms. The vase life data was then analyzed with the mixed linear model.


VL=GT+REP+STO+CT+CT:GENO+STO:CT:GENO+REP:BOX+REP:BOX:P+DV+DV:TRAY+DV:TRAY:POS+є

where *VL* is the vase life measured in days, *GT* is the temperature in the greenhouse, *REP* is the complete replication, *STO* indicates if the flower was submitted to storage conditions or not (either 0 or 1), *CT* represents the type of carnation, either standard (only one large flower on top) or spray (multiple flowers along the stem), *GENO* is the effect of each genotype, *BOX* is the blocking unit in the greenhouse, *P* is the position within the *BOX*, *TRAY* is the blocking unit in the laboratory (second phase), *POS* is the position within the *TRAY*, *DV* is the day on which the vase life assessment was started and *є* is the residual error.

The terms *GT*, *REP*, *STO*, *CT* and *CT: GENO* were analyzed as fixed factors, the remaining terms of the model were considered as random factors (**u**). We assumed a distribution $\text{u}∼N(0,\text{I}\sigma_{\text{u}}^{2})$, where $\sigma_{\text{u}}^{2}$ is the independent variance for each random term. The colon operator indicates nested effects within terms. The adjusted entry means are fitted values from the mixed linear model and were estimated with ‘ASReml-R’ version 4.2.0.267 (Butler et al., 2017) and used for further calculations. Due to the unbalance structure of the data, the *ad hoc* heritability was calculated following Piepho and Möhring (2007).

### 2.2 Genotypic data

From the 556 carnation genotypes from the trial by Boxriker et al. (2017b), 163 genotypes stemming from two full-sib carnation populations were genotyped. A total of 13,917 SNP markers were obtained through DArT sequencing (Diversity Arrays Technology, Bruce, Australia). SNP markers with more than two recorded alleles, more than 10% missing values and an expected heterozygosity below 10% were filtered out of the dataset. Individuals missing more than 10% of marker information were also left out of the analyses. After this, 7,868 SNP markers and 163 individuals, 88 individuals from population 1 and 75 from population 2, remained. This filtered data was used for all further analyses except for the linkage map construction. For map construction the SNP markers were filtered according to Taniguti et al. (2023). Sequences from the complete SNP marker dataset (13,917 markers) were initially aligned with the reference carnation genome from Zhang et al. (2022) to obtain linkage group information. Non-informative markers as well as markers missing more than 25% of data were filtered out. After filtering, SNP markers containing redundant information were allocated into bins, 5,412 bin markers and 163 individuals were retained for map construction.

### 2.3 Linkage map construction

Markers exhibiting the following segregation patterns were used for map construction: 1:1 [heterozygous for male (*aa x ab*), or for female parent (*ab x aa*)] and 1:2:1 (*ab x ab*). The recombination fractions between SNP markers were estimated with the ‘OneMap’ R package version 3.0.0 (Margarido et al., 2007; Taniguti et al., 2023). A minimum logarithm of odds (LOD) score of 7.68 and a maximum recombination fraction of 0.5 were cosidered to declare linkage between two markers. Markers for which no linkage group information was available were assigned to a linkage group based on the estimated recombination fractions. We performed an exhaustive search of the five most informative markers and their order for each linkage group. The best order was determined by the highest likelihood value. The rest of the markers were inserted in their respective linkage group according to the most likely position (Mollinari et al., 2009). Kosambi’s map funtion was used to estimate genetic distances from the recombination fractions between markers (Kosambi, 1943). Visualization of the map was rendered using the R package ‘LinkageViewMap’ version 2.1.2 (Ouellette et al., 2018).

### 2.4 QTL analysis and GWAS

After pairing the filtered SNP marker data (7,868) with the constructed linkage map, a total of 5,054 SNP markers remained for QTL analysis and GWAS. QTL analysis was carried out by implementing a composite interval mapping (CIM) model that accounts for segregation patterns from outcrossing populations based on a multipoint approach (Zeng, 1994; Gazaffi et al., 2014). For the CIM model, a maximum of 10 markers were selected as cofactors to control variation outside the mapping range and avoid over-parameterization. Cofactors were selected using multiple regression with the stepwise procedure based on the Akaike Information Criterion. The linkage map was scanned for QTL every 1 cM. A permutation test considering a window size of 15 cM and 1,000 permutations was carried out to determine a significance threshold (*α* = 0.05) according to the modification proposed by Chen and Storey (2006). The QTL analysis was carried out with the R package ‘fullsibQTL’ version 0.0.9012 (Gazaffi et al., 2014).

The GWAS was conducted with an univariate mixed linear model (Yu et al., 2006) and combining the two F1 populations, resulting in 163 genotypes and 5,054 mapped SNP markers for the analysis. The mixed linear model included principal components (PCs) as a covariate and a kinship relationship matrix (K). The PCs and K matrix were calculated from the mapped SNP markers, the K matrix was estimated according to VanRaden (2008). Only the first principal component was included into the model, since it sufficiently accounted for the subpopulation structure (**Supplementary Figure S1**). A permutation test was conducted to determine a significance threshold (*α* = 0.05). Calculations of the SNP marker scores were carried out with the R package ‘rrBLUP’ version 4.6.3 (Endelman, 2011).

### 2.5 Genomic prediction

Genomic prediction was conducted by implementing ridge regression best linear unbiased prediction (RR-BLUP) (Meuwissen et al., 2001). For the baseline model, we considered the whole set of filtered SNP markers (7,868) and their additive effects. QTL-assisted genomic prediction was tested by fitting the highest scoring SNP markers from either our QTL analyses or GWAS results (referred to as top scoring SNP markers) as fixed effects into the baseline RR-BLUP model (Bernardo, 2014; Spindel et al., 2016). The results of the QTL analyses for the two population were combined and the 120 top scoring SNP markers were selected and sorted in descending order. The 120 top scoring SNP markers from the GWAS results were also selected and sorted in descending order. We fitted the model with 1, 2, 5, 10, 25, 50, 75, 100 or 120 top scoring SNP markers as fixed effects. Additionally, the model was also fitted with both additive and dominance effects of the SNP markers and compared to a model with only additive effects. The model was evaluated as:


$$y=Xb+{\text{Z}}_{a}a+{\text{Z}}_{d}d+є$$

where **y** is a vector of adjusted entry means for vase life, **b** is a vector of fixed effects for the top scoring SNP markers fitted to the model, a is a vector of random additive effects, d is a vector of random dominance effects and *є* is a vector of residual errors. **X, Z_a_** and **Z_d_** are incidence matrices relating, b, a and d to the phenotypic records. Xb includes an intercept 1*_n_µ*. The encoding of the SNP markers in **Z_a_** and **Z_d_** are [-1, 0, 1] and [0, 1, 0] for the genoytypes *A*_1_*A*_1_*,A*_1_*A*_2_*,A*_2_*A*_2_, respectively (Vitezica et al., 2013). We assumed distributions of $a∼N(0,I\sigma_{d}^{2})$, $d∼N(0,I\sigma_{d}^{2})$, $є∼N(0,I\sigma_{є}^{2})$, where $\sigma_{a}^{2}$, $\sigma_{d}^{2}$ and $\sigma_{є}^{2}$ are the additive genetic variance, dominance genetic variance and the residual variance, respectively. I is an identity matrix. ${\text{Z}}_{d}d$ was left out when modelling only additive effects.

The prediction accuracy of the fitted models was assessed through cross-validation. For each of 1,000 cross-validation iterations, the dataset was randomly split 80:20 into training and validation sets. Pearson’s correlation between observed phenotypic values *y* and predicted phenotypic values $\hat{y}$ from the validation set was employed as the measurement of prediction accuracy $r(y,\hat{y})$. We used the R package ‘sommer’ version 4.3.4 (Covarrubias-Pazaran, 2016) to run the genomic prediction models.

## 3 Results

### 3.1 Evaluation of vase life

Adjusted entry means for vase life were calculated for two F1 carnation populations from a two-phase one-year trial. Vase life ranged between 5.6 and 16.91 days, with an average of 10.17 days. The estimated *ad hoc* heritability was 0.74. A detailed description of the vase life trial results is provided in Boxriker et al. (2018).

### 3.2 Linkage map construction

**Figure 1:**
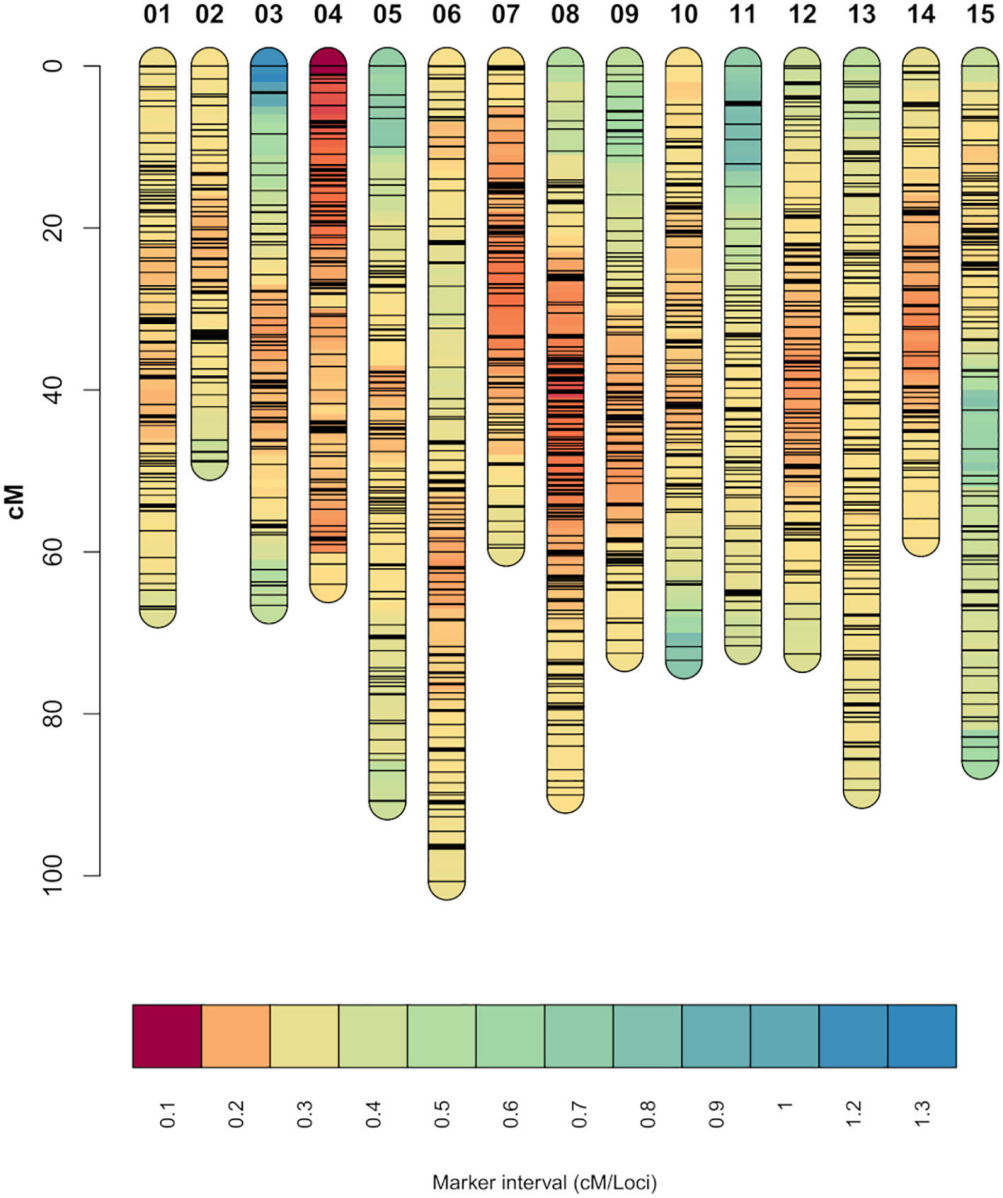
Genetic linkage map constructed from the two experimental carnation populations. Shorter marker intervals indicate areas of higher marker density. Each black horizontal line on the individual chromosomes represents a SNP marker.

From the 13,917 SNP markers obtained by DArT sequencing, 11,896 SNP markers could be assigned to one of the 15 chromosomes of carnation based on the reference genome from Zhang et al. (2022). After filtering out markers for missing data and non-informative markers, 8,609 SNP markers were retained. The SNP markers were then grouped into 5,412 bin markers for map building, with an average of 1.59 SNP markers per bin. The integrated genetic information of the bin markers resulted in a genetic map spanning 1,116.16 cM (**Figure 1**). The number of SNP markers per linkage group ranged from 230 to 539, belonging to linkage groups 2 and 8, respectively. The average marker density was 4.93 loci/cM and the average marker interval 0.21 cM/loci. The lowest and highest marker densities, 3.32 and 7.31 loci/cM, belonged to linkage groups 11 and 4, respectively. The longest marker interval distance was 16.83 cM located on linkage group 3 (**Table 1**). Correlation between the markers’ physical positions on the reference genome and their positions on the constructed genetic map was evaluated (**Supplementary Figure S2**). In general, the marker order on the genetic map was consistent with that on the reference genome. The average Pearson’s correlation coefficient between genetic and physical positions for the 15 linkage groups was 0.85. All linkage groups had a correlation coefficient above 0.7, with the lowest and highest correlation coefficients belonging to linkage groups 1 and 8, 0.73 and 0.93 respectively (**Table 1**).

**Table 1:** Summary of the constructed linkage map.

Linkage group	No. markers	Length (cM)	Density (loci/cM)	Average distance (cM)	Correlation
1	335	67.10	4.99	0.2	0.73
2	230	48.91	4.7	0.21	0.84
3	278	66.62	4.17	0.24	0.87
4	468	63.96	7.32	0.14	0.89
5	368	90.79	4.05	0.25	0.9
6	493	100.72	4.89	0.2	0.89
7	382	59.54	6.42	0.16	0.92
8	539	89.97	5.99	0.17	0.93
9	343	72.48	4.73	0.21	0.89
10	348	73.41	4.74	0.21	0.92
11	238	71.59	3.32	0.3	0.88
12	379	72.62	5.22	0.19	0.76
13	389	89.35	4.35	0.23	0.86
14	330	58.33	5.66	0.18	0.8
15	292	85.78	3.4	0.29	0.77

Correlation as Pearson’s correlation between genetic and physical positions.

### 3.3 QTL analysis and GWAS

**Figure 2:**
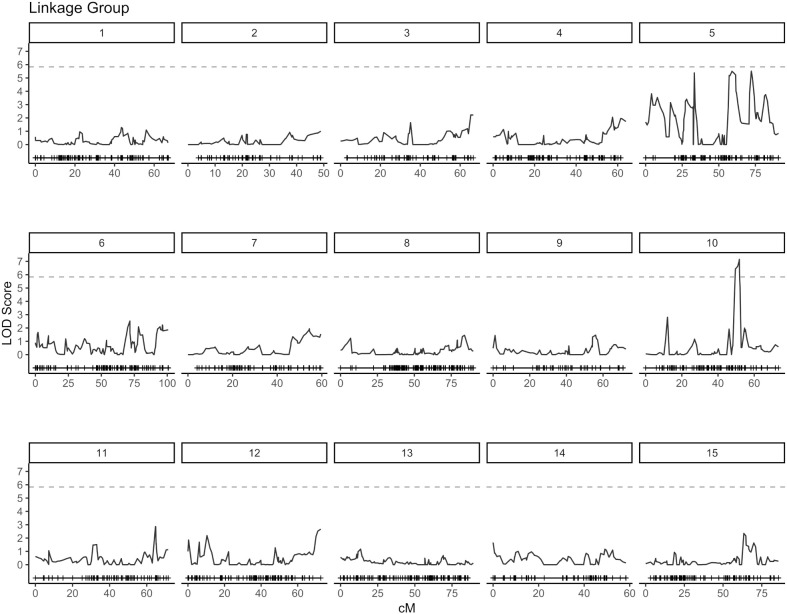
Composite interval mapping plot for population 1. Phenotypes of 88 individuals and 5,054 SNP markers. Significance threshold (*α* = 0.05) at LOD = 5.83 from a permutation test.

**Figure 3:**
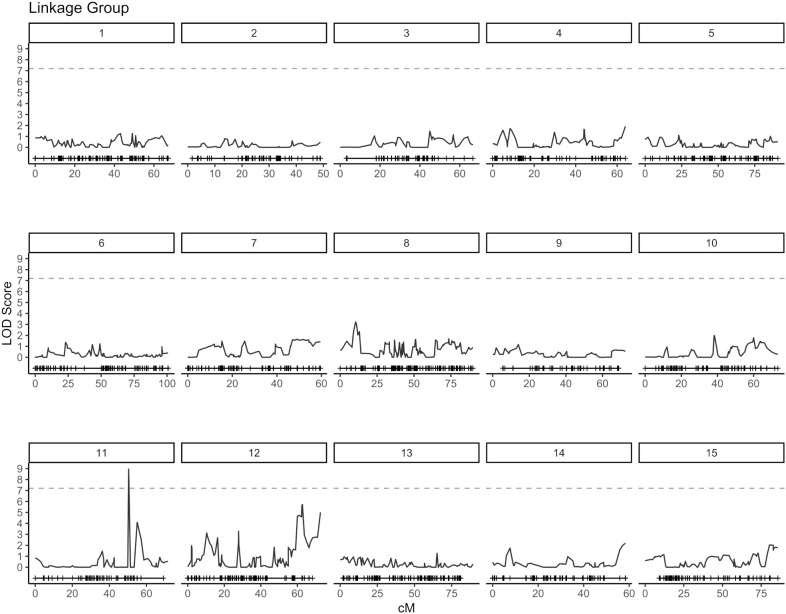
Composite interval mapping plot for population 2. Phenotypes of 75 individuals and 5,054 SNP markers. Significance threshold (*α* = 0.05) at LOD = 7.19 from a permutation test.

QTL analysis for vase life was carried out with the constructed genetic linkage map and composite interval mapping. Two F1 populations were evaluated independently. Population 1 consisted of 88 individuals and population 2 of 75 individuals. For population 1, a significance threshold (*α* = 0.05) was set at a LOD score of 5.83 by a permutation test. A significant QTL signal with LOD score of 7.16 was observed on linkage group 10 between 49.45 and 51.77 cM (**Figure 2**). The SNP marker *M3001191510* was located at the peak of this signal. The proportion of variance explained by this SNP marker was 5.09%. Additionally, three high scoring SNP markers were also detected on linkage group 5. These signals did not exceed the significance threshold but are described in **Table 2**. For population 2, a significance threshold (*α* = 0.05) was set at a LOD score of 7.19 by a permutation test. One significant QTL signal was detected (**Figure 3**). The SNP marker *M3000121965*, located at 50.39 cM in linkage group 11, had a LOD score of 8.94 and explained 2.84% of the phenotypic variance.

**Table 2:** Summary significant SNP markers detected by CIM for both F1 populations.

Population	SNP ID	Linkage group	Position (cM)	MAF	LOD score	PVE (%)	A
pop1	M2999911757	5	33.26	0.48	5.38	0.37	1.23
pop1	M3001144616	5	58.99	0.27	5.50	0.53	-0.66
pop1	M3001744512	5	72.22	0.49	5.52	1.87	-0.44
pop1	M3001191510	10	51.77	0.40	7.16	5.09	1.88
pop2	M2999852250	11	50.39	0.19	8.94	2.84	-0.90

*MAF* Minor allele frequency; *PVE* phenotypic variation explained; *A* phenotypic effect.

The GWAS was carried out based on a univariate mixed linear model with the constructed genetic linkage map and both populations combined. A significance threshold (*α* = 0.05) was set at a -log_10_(*p*) score of 4.31 by a permutation test. None of the tested SNP markers exceeded the significance threshold. However, SNP marker peaks could be observed on 10 out of the 15 linkage groups (**Supplementary Figure S3**). The top scoring SNP markers were located on linkage groups 10 and 13. On linkage group 13, a group of 14 SNP markers located between 80.45 and 83.57 cM had -log_10_(*p*) scores ranging from 2.02 to 3.27. The estimated additive effects for these 14 SNP markers were all positive and ranged from 0.58 to 1.72 days. On linkage group 10, a high scoring SNP marker was observed at 47.43 cM. It had an estimated additive effect of -1.55 days. The variance explained by these SNP markers did not exceed 5%. The top scoring SNP markers on the remaining linkage groups are described in **Supplementary Table S1**.

### 3.4 Genomic prediction

**Figure 4:**
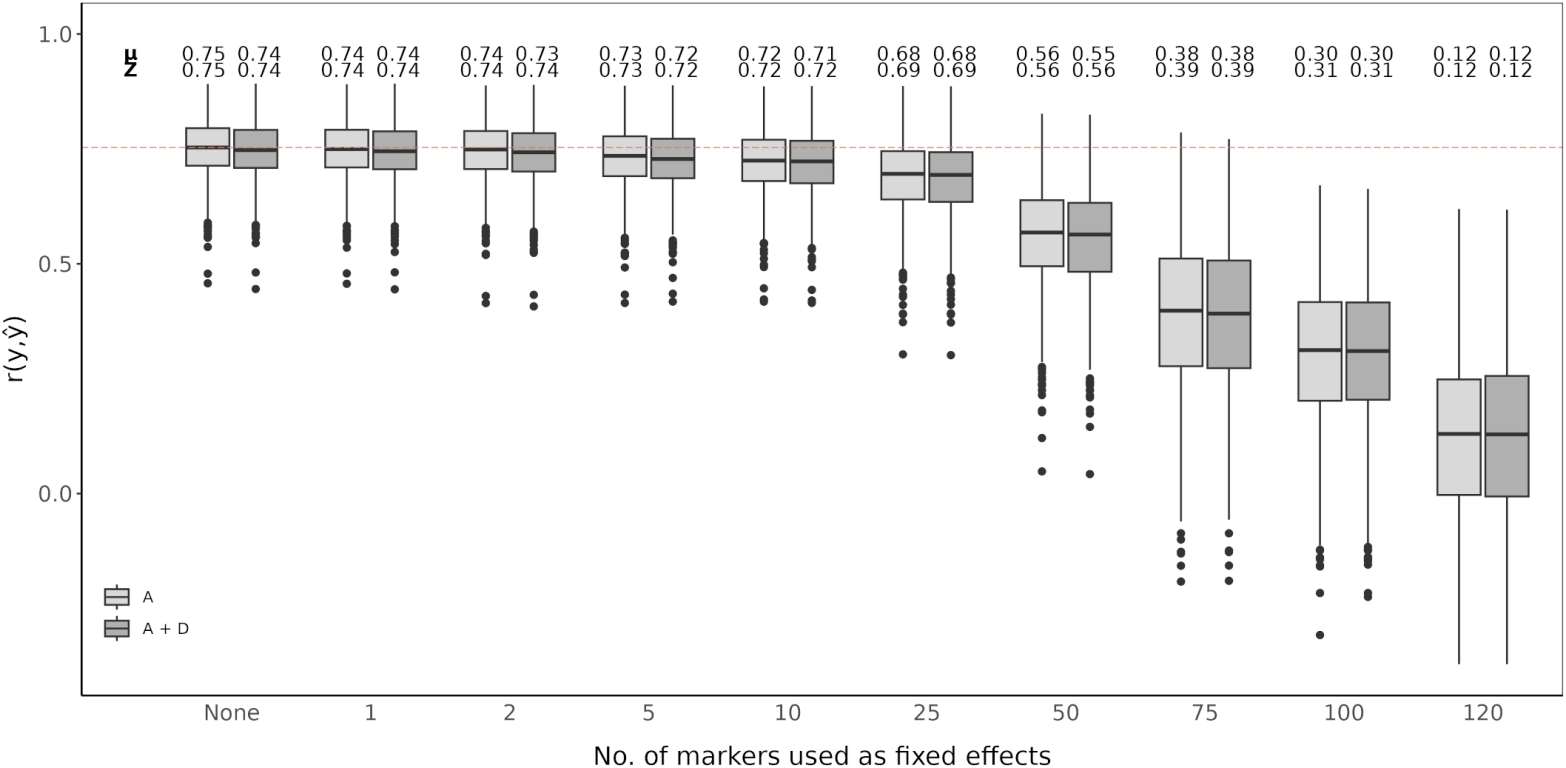
Genomic prediction of vase life with a different amount of top scoring QTL analysis SNP markers included as fixed effects. The boxplots show the correlation $r(y,\hat{y})$ between the observed phenotypic values *y* and the predicted phenotypic values $\hat{y}$ in the validation set for 1,000 cross-validation runs. All models were evaluated for additive effects (light gray; A) or additive plus dominance effects (dark gray; A + D). Mean (*µ*) and median (Z) of the 1,000 runs of each model above the respective boxplot.

**Figure 5:**
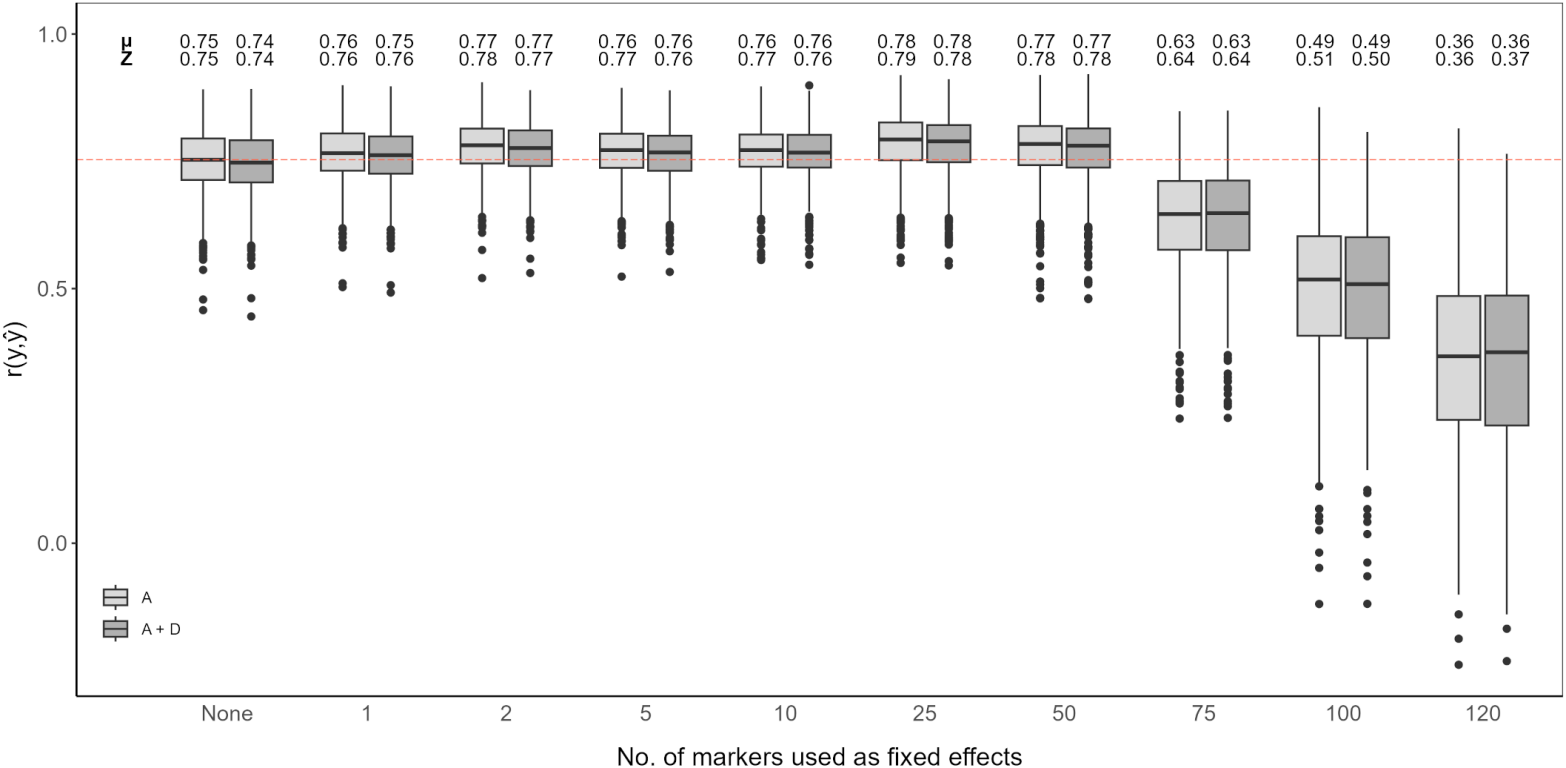
Genomic prediction of vase life with a different amount of top scoring GWAS SNP markers included as fixed effects. The boxplots show the correlation $r(y,\hat{y})$ between the observed phenotypic values *y* and the predicted phenotypic values $\hat{y}$ in the validation set for 1,000 cross-validation runs. All models were evaluated for additive effects (light gray; A) or additive plus dominance effects (dark gray; A + D). Mean (*µ*) and median (Z) of the 1,000 runs of each model above the respective boxplot.

An RR-BLUP based genomic prediction model was evaluated and fitted with top scoring SNP markers as fixed effects. A model without added top scoring SNP markers considering only additive effects was used as the baseline (**Figures 4**, **5**). Prediction accuracy was measured as the average of the correlations between observed and predicted values ( $r(y,\hat{y})$). The baseline model showed an average prediction accuracy of 0.75 over the 1,000 cross-validation iteration. Considering both additive and dominance effects, without adding any top scoring SNP markers, resulted in an average prediction accuracy of 0.74. Adding top scoring SNP markers detected by QTL analysis did not increase prediction accuracy. In general, the prediction accuracy further declined the larger the amount of top scoring SNP markers added. This tendency was observed for models considering only additive affects as well as additive and dominance effects (**Figure 4**).

Adding top scoring SNP markers detected by GWAS improved prediction accuracy. In both cases, considering only additive effects or additive and dominance effects together, prediction accuracy increased when adding between 1 and 50 top scoring SNP markers as fixed effects. The highest prediction accuracy was observed when including 25 top scoring SNP markers for only additive effects, with an average correlation of 0.78. Beyond 50 top scoring SNP markers, there was a decline in the prediction accuracy, the lowest being for 120 added top scoring SNP markers at 0.36 (**Figure 5**).

## 4 Discussion

### 4.1 Linkage map for vase life in carnation

A widely used approach for dissecting complex traits in crop plants is QTL analysis. An essential part for this is the availability of a genetic map. In this study, we constructed a genetic map based on 5,412 SNP markers and spanning 1,116.16 cM (**Figure 1**). Previously reported genetic maps for *D. caryophyllus* were built either using SNP markers together with simple sequence repeats (SSR) (Yagi et al., 2013) or only using SSR markers (Yagi et al., 2017). To our knowledge, this is the first map for *D. caryophyllus* based on SNP bin markers. A bin map algorithm reduces the chance of having false-positive markers in the map by removing redundant markers for the map building process (Liu et al., 2023). The present genetic map comprises a larger amount of marker information than previously published maps, while spanning a relatively similar distance compared to 969.6 cM (Yagi et al., 2013) and 971.5 cM (Yagi et al., 2017). The marker density of the constructed map is higher than that of previously reported carnation genetic maps (Yagi et al., 2017). Moreover, the chromosome information used to arrange the SNP markers was obtained from a reference genome with a higher benchmarking universal single-copy orthologs (BUSCO) integrity than that of previous reference genomes (Zhang et al., 2022). This, together with the good coverage of the chromosomes, makes the present genetic linkage map an effective tool for studying the genetic basis of vase life in carnation.

### 4.2 Genetic architecture of vase life in carnation

An understanding of the genetic architecture of a trait requires knowledge about the number and genome locations of genes influencing the phenotypic expression of the trait. This can be assessed through QTL analysis and GWAS (Mackay, 2001). In this study, composite interval mapping was carried out for two biparental carnation populations separately. For each population, signals above the significance threshold were observed, indicating the presence of potential QTL for vase life. However, the positions of relevant signals did not show an overlap for the two populations. For population 1, a QTL signal was observed on linkage group 10 between 49.45 - 51.77 cM, while for population 2 one QTL signal occured on linkage group 11 at 50.39 cM (**Figures 2**, **3**). Furthermore, at the positions where significant signals were observed for one population, there was little to no signal for the other. Incongruent signals in QTL analyses between different crosses have been reported by other authors (Pilet et al., 2001; Mihaljevic et al., 2004), where they have been mostly attributed to small population sizes. Smaller populations in QTL analysis tend to increase the error rate in estimating QTL number, position and effects (Beavis et al., 1991). Additionally, it is likely that the two F1 populations in this study represent distinct genetic pools (**Supplementary Figure S1**). This would imply that different genetic resources are being leveraged for each population, which could be valuable for broadening the genetic base for breeding, and also explain the inconsistent signals between the two populations.

In general, research exploiting genomic resources is still lacking in ornamental crops compared to cereal and other crops. To our knowledge, QTL analysis for vase life in carnation has not been investigated so far. However, it has been investigated in other ornamentals, like lily (*Lilium* L.) (Pourbeyrami Hir et al., 2019) and rose (*Rosa* × *hybrida*) (Carvalho et al., 2015). In lily the authors could identify two QTL explaining variation of vase life. Similar to our case, the effect sizes of these QTL were moderate, which the authors attributed to small population sizes (Pourbeyrami Hir et al., 2019). In rose, a QTL analysis for stomatal function, which is a determinant in post-harvest longevity, reported several QTL regions explaining both major and minor amounts of variation in stomatal function (Carvalho et al., 2015).

A strategy to overcome issues in covered allelic diversity as well as mapping resolution power is the joint analysis of multiple populations (Holland, 2007; Meng et al., 2016). On this basis, we conducted a GWAS with the two populations merged into one. The GWAS was carried out using the combined population, while accounting for population structure. From our results, we observed a large number of markers with small effect sizes across 10 out of the 15 chromosomes. This follows the standard quantitative model, where QTL are distributed, for the most part, evenly along the genome (**Supplementary Figure S3**).

Even with the advent of next-generation sequencing, GWAS in ornamentals are still rare in comparison to other crops, and for vase life there are no reports of them so far. Conversely, senescence as a trait has been more widely investigated in different plant species (Harris et al., 2007; Thomas and Ougham, 2014; Hassan et al., 2021). Although vase life and plant senescence are two separate traits, they share common mechanisms, like production of and tolerance to stress hormones, which makes them comparable to a certain extent (Rogers, 2013). The genetic architecture of senescence in crop plants has been described as polygenic, without entirely following the standard quantitative model, to highly quantitative depending on the crop (Yannam et al., 2022; Chapman et al., 2021). In maize, a review of quantitative genetics studies investigating senescence reported 85 QTL located along all 10 chromosomes, primarly on chromosomes 1 and 5, and explaining between 2.4 to 25% of phenotypic variance of the trait (Munaiz et al., 2020). More specifically, 46 QTL each explainig between 1 to 10% of the variance for senescence were identified in maize by a GWAS (Yannam et al., 2022). Based on their results, the authors argue that the genetic architecture of senescence follows that of a polygenic trait, although QTL associated with the trait are not evenly distributed across the genome. Senescence in upland cotton has also been investigated through a multi-locus GWAS (Liu et al., 2022). The study identified 50 genomic regions associated to senescence, located on 22 out of the 26 pairs of chromosomes of cotton. From their results, they concluded that senescence in cotton is controlled by a highly complex regulatory mechanism, corroborating the quantitative nature of the trait. As shown for senescence, the genetic architecture of vase life possibly varies in complexity depending on the crop. For carnation, bulked segregate analysis (BSA) has evidenced a total of 229 differentially expressed genes between varieties with short and long vase life (Boxriker et al., 2017a). This suggests that there are numerous molecular mechanisms involved in determining how long lasting carnation varieties can be (Tanase, 2020b).

Through QTL analysis, we detected one potential QTL for each of the two populations studied, on chromosomes 10 and 11 for populations 1 and 2, respectively. However, further validation of these results is needed through the use of larger populations to improve statistical power and mapping resolution (Li et al., 2007). By combining the two F1 populations, we could detect through GWAS further QTL signals in other genomic regions, the strongest of them being on chromosome 13. The intensity of these QTL signals however is insufficient to consider them causal variants. Instead, these signals provide a preliminary view of the genetic architecture underlying vase life. This architecture appears to resemble the pattern of senescence in maize, with polygenic inheritance, though not as highly quantitative as traits such as flowering time (Yannam et al., 2022; Lakmes et al., 2022). We acknowledge however, that F1 populations are not ideal for a GWAS. Due to the limited number of segregating alleles and recombination events, these populations harbor limited genetic diversity. Even after combining the two populations, which broadened the genetic base for the analysis, we did not observe significant QTL signals and a more genetically diverse panel would be needed to carry out a robust GWAS.

### 4.3 Improving vase life through genomic selection

Genomic selection is regarded as a powerful breeding tool for genetic gain in quantitative traits (Bernardo and Yu, 2007; Spindel et al., 2015; Crossa et al., 2017). So far there have been no studies applying genomic prediction to improve vase life in ornamental crops. Nevertheless, genomic selection has been proposed as potentially useful for rose breeders, particularly in traits like senescence, scent and plant architecture, among others (Smulders et al., 2019). Additionally, in carnation, as is the case for many perennial crops, long generational times make the breeding of target traits a long endeavor, which means that conventional breeding programs can carry on for decades to produce new commercial varieties (Onozaki, 2018). In coffee, studies have suggested that implementing genomic selection to growth, production and pathogen resistance related traits could reduce the conventional 6-year breeding cycle to a 3-year one (Seyum et al., 2022). In sugar cane, another important perennial crop, genomic prediction has been applied to traits like fiber content and yield (Hayes et al., 2021). Although for both traits prediction accuracy was moderate, 0.3 and 0.44 respectively, the authors emphasize the improvement in genetic gain through shorter breeding cycles (Deomano et al., 2020; Hayes et al., 2021). Here, we observed a high prediction accuracy for vase life in carnation. This result is consistent with the reported heritability for vase life in this trial (0.74) by Boxriker et al. (2018) and provides evidence that genomic selection could increase the genetic gain of this trait (Crossa et al., 2014).

Estimation of non-additive effects can produce more accurate estimates of breeding values (Toro and Varona, 2010). In clonally propagated crops, including dominance effects in genomic prediction models has improved prediction accuracy (Seyum et al., 2022; Werner et al., 2023). For fruit quality traits in strawberry, a GBLUP approach that accounts for both additive and dominance effects showed a slight improvement in prediction accuracy compared to a model including only additive effects (Yamamoto et al., 2021). In our case, accounting for dominance effects along side the additive effects did not seem to have an impact on the prediction accuracy. In many cases, inclusion of dominance effects in genomic prediction models only negligibly improves prediction accuracy, and reliable statistical testing to identify the advantage of dominance effects requires larger more comprehensive datasets (Varona et al., 2018).

Integration of both historical and new QTL information into genomic prediction models has been investigated in crop plants to improve prediction accuracy (Bernardo, 2014; Spindel et al., 2016; Rice and Lipka, 2019; Sehgal et al., 2020). In rice, extending the genomic prediction RR-BLUP model by including top scoring GWAS markers was shown to improve prediction accuracy (Spindel et al., 2015, 2016). The authors observed increased prediction accuracy for quantitative traits, like grain yield and plant height. Additionally, they compared a GWAS-assisted genomic prediction approach based on historical data, where the selected markers included in the genomic prediction model came from already known functional markers, with a *de novo* GWAS approach, where selected markers came from a GWAS carried out with the same marker information. Their results showed that the *de novo* approach further increased prediction accuracy compared to using historical information (Spindel et al., 2016). This is particularly favorable for genomic prediction studies in ornamentals, since the amount of available historical information on functional markers is not as extensive as for other crops. A GWAS-assisted genomic prediction approach can also be beneficial when having a small population size, as with this approach, small populations tend to be sufficient to accurately estimate major QTL effects fitted as fixed effects (Bernardo, 2014). This is common in ornamental crop research, where sample populations are usually small (Van Huylenbroeck, 2018), although, as reported for *Populus cathayana*, very small population sizes can result in insufficient GWAS signal intensity for a GWAS-assisted genomic prediction approach to be effective (Zhou et al., 2023). From our QTL analysis and GWAS, we could not identify QTL explaining large amounts of variation. We could detect significant QTL signals with CIM for both populations, however fitting these detected SNP markers to the RR-BLUP model did not show an improvement in the prediction accuracy (**Figure 4**). In contrast, even with relatively weak GWAS signal intensities in our data, we could observe an increase in prediction accuracy with a GWAS-assisted genomic prediction approach (**Figure 5**). The improvement provided by the GWAS-derived SNP markers may be due to their better reflection of the trait’s polygenic nature, compared to the SNP markers identified through separate QTL analyses of the populations.

The optimal number of top scoring SNP markers in the genomic prediction model depends on the genetic architecture of the trait (Bernardo, 2014). A simulation study showed that, for a trait with many minor QTL, fitting between 10 and 100 QTL to an RR-BLUP model significantly increased the prediction accuracy in comparison to models in which only 1 to 5 QTL were fitted. Conversely, simulating few major QTL with large effects resulted in mean prediction accuracies that were relatively similar, regardless of the number of QTL added as fixed effects (Rice and Lipka, 2019). A study in chrysanthemum, with a marker dataset of over 300,000 SNP markers, showed that when adding the top 500 and top 1,000 SNP markers detected by a GWAS as fixed effects to the RR-BLUP model, prediction accuracy for plant height increased (Zhang et al., 2024). However, when fitting only the 42 significantly associated SNP markers as fixed effects, the increase in prediction accuracy was minor. In contrast, for flowering time related traits, with the same chrysanthemum dataset, the highest prediction accuracies were reported when using the top 100 SNP markers or less (Su et al., 2024). In our case, fitting the first 1 to 50 top scoring GWAS SNP markers as fixed effects improved the genomic prediction model while adding more markers beyond this point drastically deteriorated prediction accuracy for vase life. This decrease in accuracy could originate from model over-fitting, when including further SNP markers as fixed effects, the estimated marker effects become less reliable to predict the performance on the validation set. It is likely that for other relevant traits in cut carnation, like stem length or flower count, the optimal number of top scoring SNP markers differs from that observed for vase life. The optimal number of SNP markers to maximize prediction accuracy should be determined on a trait-to-trait basis, where the genetic architecture of the trait, and possibly the size of the dataset, are taken into account.

Our findings contribute to the better understanding of the genetic architecture of vase life as well the potential of genomic selection for this trait. While here we observed a high mean prediction accuracy for the baseline RR-BLUP model (0.75). The use biparental populations might have artificially inflated this value, due to high relatedness between individuals (Lorenz and Smith, 2015). A more accurate estimate of the prediction accuracy for vase life could be determined by having a larger and more diverse training population together with a larger marker dataset (Hickey et al., 2014). Nonetheless, our results suggest that genomic selection could accelerate breeding of longer lasting cut carnation varieties. Additionally, extending a genomic prediction approach with GWAS results can help to further utilize the information present in a given genotypic dataset (Spindel et al., 2016). This can be of special interest for the study of non-major crops, like ornamentals, where the availability genetic marker data can be limited.

## Data Availability

The data presented in this study are publicly available. The data can be found in GitHub (https://github.com/hhtaveras/vaselife_carnation) and Zenodo (10.5281/zenodo.16311429).
