# Supplementary material for: Genetic architecture and genomic prediction of vase life in carnation
Source: Front Plant Sci. 2025 Dec 5;16:1673111. doi: 10.3389/fpls.2025.1673111 (PMC12714970; doi:10.3389/fpls.2025.1673111)
Supplement: Supplementary file 1 [file DataSheet1.pdf]

Supplementary Material

1 SUPPLEMENTARY TABLES AND FIGURES

1.1 Figures

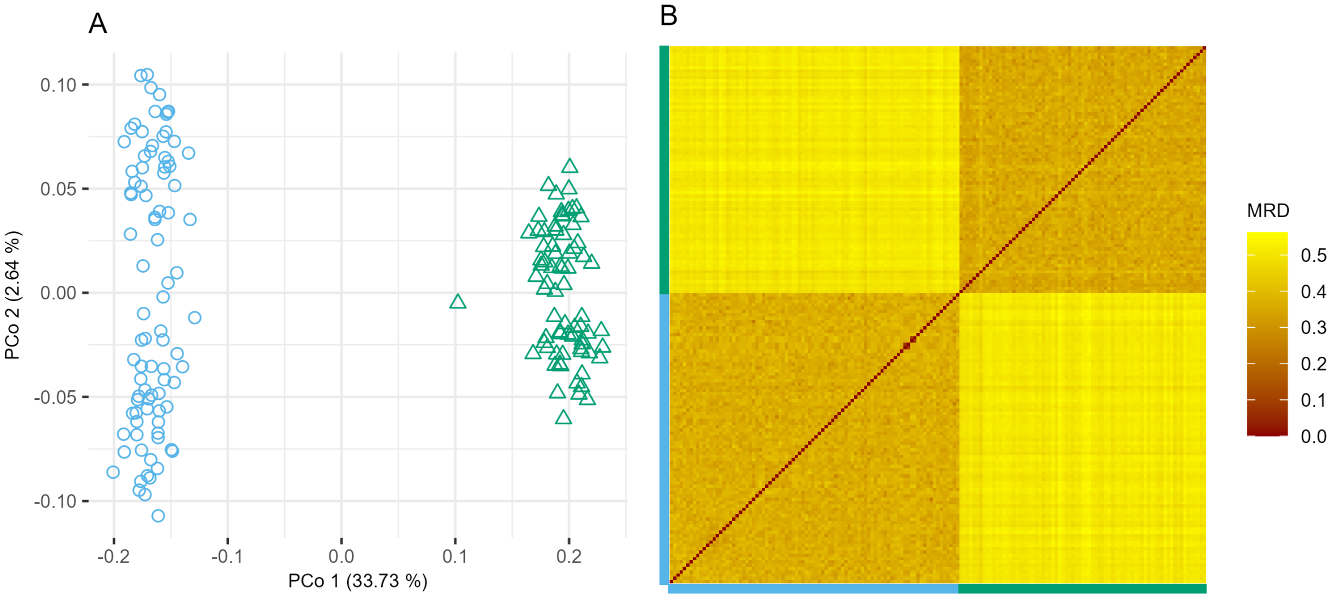

Figure S1. Population structure for both F1 populations; population 1 (green), population 2 (blue). **(A)** Principal coordinates analysis based on the pairwise Roger's modified distances (MRD) between individuals of both populations. Proportion of variance explained by the first (PCo 1) and second (PCo 2) principal coordinates. **(B)** Heatmap showing the distances between individuals of the two experimental carnation populations.

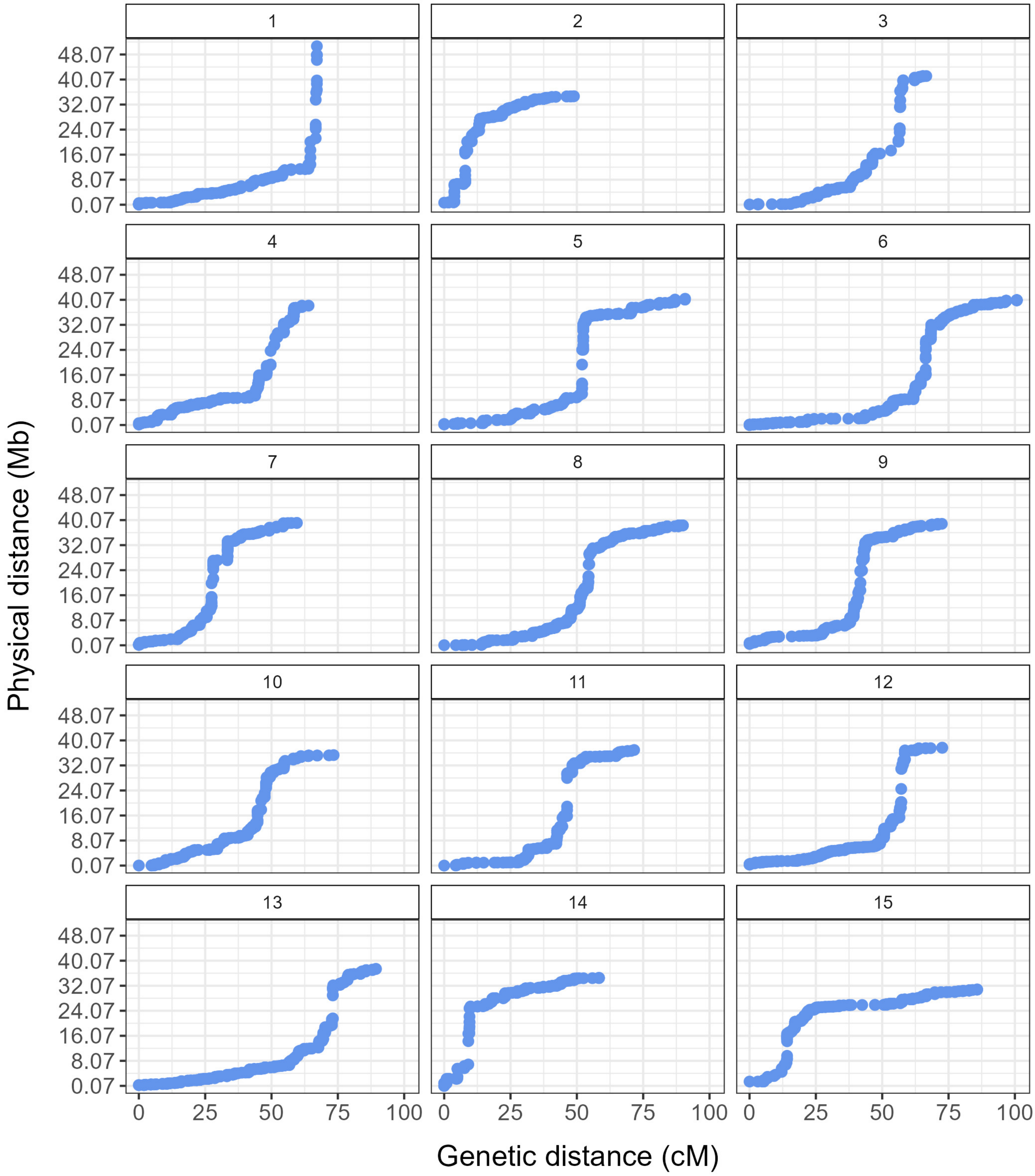

Figure S2. Correlation between the genetic and the physical positions of the 5,412 mapped SNP markers on their respective linkage group.

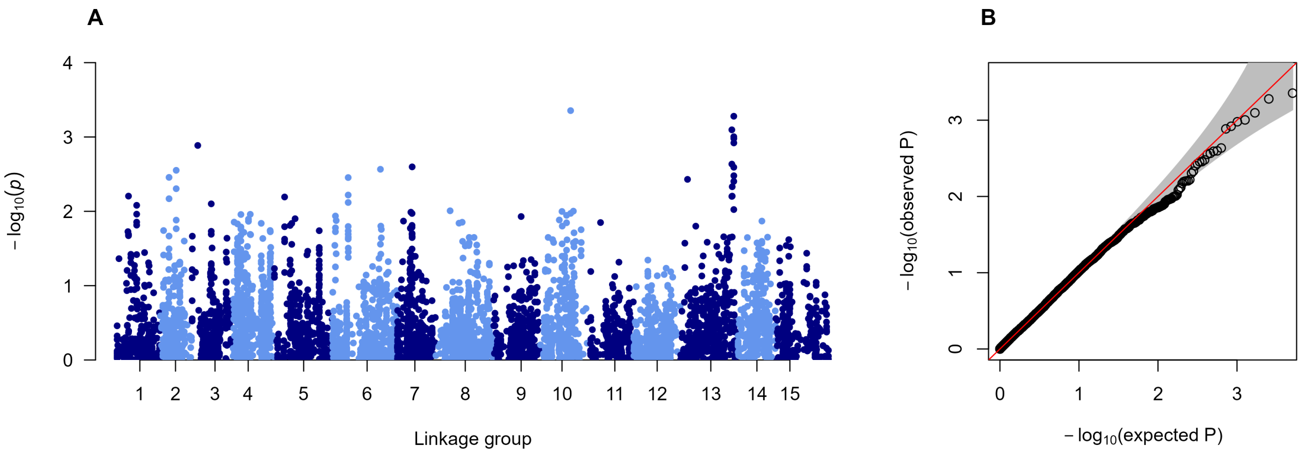

Figure S3. GWAS for vase life based on a univariate mixed linear model. **(A)** Manhattan plot showing association of SNP markers based on (-$\log_{10}(p)$) and their respective linkage group. **(B)** QQ plot showing observed (-$\log_{10}(p)$) versus expected (-$\log_{10}(p)$) values.

1.2 Tables

Table S1. List of SNP markers identified as significant from GWAS. *MAF* Minor allele frequency; *PVE* phenotypic variation explained; *A* phenotypic effect

SNP ID	Linkage group	Position (cM)	MAF	$-log_{10}(p)$	PVE (%)	A
M3001929420	1	17.86	0.41	2.20	1.93	0.95
M3001858406	1	30.71	0.29	2.08	0.07	-0.79
M2999848630	2	13.54	0.14	2.46	2.17	-1.33
M3000152914	2	13.54	0.13	2.17	2.10	-1.26
M3000746529	2	24.81	0.40	2.55	1.49	0.81
M3000656447	2	24.81	0.39	2.30	0.04	0.75
M3000876524	3	11.97	0.15	2.89	3.53	-1.60
M3001268550	3	33.19	0.31	2.10	3.19	-1.00
M2999995542	5	16.01	0.08	2.19	0.28	1.27
M3001939823	6	24.24	0.35	2.12	0.40	-0.64
M3002041239	6	24.24	0.28	2.22	0.12	-0.94
M3001241230	6	24.24	0.29	2.45	0.07	-0.98
M3000150034	6	74.95	0.29	2.57	3.70	-1.04
M3000700716	7	23.14	0.14	2.60	1.55	-1.42
M3001861418	8	22.33	0.25	2.01	0.27	-0.90
M3001719011	10	47.43	0.13	3.35	2.14	-1.54
M3000010344	10	51.77	0.48	2.00	2.28	-1.08
M3001366531	13	10.71	0.41	2.43	2.08	0.86
M2999906118	13	80.45	0.24	3.10	0.00	1.09
M3000630737	13	80.45	0.24	2.20	0.85	1.01
M3000917239	13	80.45	0.24	2.64	0.50	1.04
M3000769819	13	81.01	0.24	2.33	0.04	1.00
M3000387456	13	81.01	0.37	2.20	1.16	0.58
M3001100722	13	83.51	0.16	2.02	0.03	0.81
M2999855144	13	83.51	0.12	3.28	0.14	1.72
M3000279945	13	83.51	0.12	2.40	0.31	1.41
M3000967931	13	83.51	0.19	2.59	0.01	0.83
M2999931043	13	83.51	0.12	3.00	0.00	1.52
M2999847964	13	83.57	0.13	2.98	4.05	1.36
M3000122923	13	83.57	0.12	2.92	0.07	1.63
M3001870031	13	83.57	0.17	2.48	0.06	0.92
